# Stability of arterial blood gas samples after delayed analysis and mechanical stress

**DOI:** 10.1371/journal.pone.0334710

**Published:** 2025-12-04

**Authors:** Max Gutermuth, Harald Ihmsen, Frederick Krischke, Andreas Moritz, Johannes Prottengeier

**Affiliations:** 1 Department of Anesthesiology, Erlangen University Hospital, Erlangen, Germany; 2 Faculty of Medicine, Friedrich Alexander University Erlangen Nuremberg, Erlangen, Germany; 3 Outpatient Surgery Center Bernard and Colleagues, Erlangen, Germany; 4 Department of Anesthesiology and Critical Care, Klagenfurt Hospital, Klagenfurt, Austria; KIST: Korea Institute of Science and Technology, GERMANY

## Abstract

**Purpose:**

To investigate the effect of prolonged time before analysis and mechanical manipulation on pre-analytical stability of biomarkers and the validity of blood gas analysis results.

**Methods:**

We collected blood samples from 240 ICU patients from May 18, 2022 to March 31, 2023. Samples were analyzed immediately per standard operating procedure, then the syringes were kept at room temperature for 60 min, subjected to standardized mechanical forces (repeated drops) and analyzed again. Thirteen typical blood gas analytes were measured. Bland–Altman plots were prepared to assess differences between initial and delayed analyses. Differences were compared against official accuracy limits specified in German quality assurance guidelines (Rili-BAEK).

**Results:**

For hemoglobin, creatinine, glucose, and electrolytes (calcium, sodium, chloride, bicarbonate), agreement between immediate and post-delay analyses remained within the official acceptable ranges. For pH and potassium, deviations exceeded the Rili-BAEK accuracy limits but remained clinically acceptable. Only oxygen partial pressure and lactate levels changed so markedly that they would no longer be reliable for clinical interpretation.

**Conclusion:**

Even after a 60-min delay and excessive mechanical stress, selected blood gas analytes such as hemoglobin, glucose, and electrolytes can be considered valid. Potassium and carbon dioxide partial pressure were altered but might be suitable for approximation purposes. Findings for oxygen partial pressure and lactate were generally invalid. In the future, these findings can aid in reducing unnecessary blood sampling. These findings may guide clinicians in deciding whether repeat sampling is necessary, potentially reducing unnecessary blood draws, while reinforcing that critical parameter (pO₂, pCO₂, pH) still require prompt analysis.

## Introduction

In anesthesia and intensive care medicine, blood gas analysis is a basic diagnostic tool for at-risk and critically ill patients. Careful handling of samples after collection and immediate analysis are accepted standards. Nevertheless, numerous situations in everyday clinical practice can lead to unintentional analysis delays or mechanical interference with samples. Such situations include lengthy transport routes, equipment downtime for maintenance, human error, or mechanical shocks from mishandling. The current literature does not definitively answer whether samples remain suitable for analysis after prolonged periods and/or mechanical interference. This uncertainty often prompts clinicians to obtain new blood samples, incurring costs, time, and potential patient discomfort and harm associated with renewed blood loss or repeated vascular punctures [1,2]. In critical care settings, arterial blood gas tests are among the most frequently performed diagnostic blood tests, accounting for up to ~40% of diagnostic blood loss. ICU patients have been reported to undergo a median of 8 blood gas analyses per day, resulting in approximately 45 mL of blood loss daily from arterial sampling. Such cumulative phlebotomy can contribute to iatrogenic anemia and increase the need for transfusions. Therefore, avoiding unnecessary repeat blood gas draws is important for patient safety and resource stewardship. Existing literature has only focused on certain aspects of pre-analytical stability of blood gas samples. For example, Knowles and Harsten observed changes in respiratory gas tensions (pO₂ and pCO₂) 30 and 60 minutes post-collection. Smeenk et al. [3] described prolonged pO₂ stability when using glass syringes or ice-water storage. However, beyond respiratory gases, modern blood gas analyzers measure various clinically relevant analytes of ionic or small-molecule character (e.g., sodium, potassium, calcium, glucose, lactate), and data on the pre-analytical stability of these numerous parameters are limited. In a recent study, we demonstrated that time delays and mechanical stress on venous blood samples in an out-of-hospital emergency setting did not lead to any clinically relevant alterations in various electrolytes, small organic molecules, or protein biomarkers [4]. Additionally, laboratory guidelines advise that samples in plastic syringes be kept at room temperature and analyzed as soon as possible (ideally within 15–30 minutes) to maintain blood gas stability. It is known that many routine analytes (e.g., electrolytes and metabolites) remain relatively stable for short periods, whereas pO₂, pCO₂, and lactate can change significantly due to ongoing cellular metabolism and gas exchange. However, comprehensive evidence encompassing the full panel of blood gas analytes under realistic ICU conditions is lacking. Therefore, this study aimed to investigate the effect of prolonged delay before analysis and mechanical manipulation on the pre-analytical stability of arterial blood gas samples and the validity of the analysis results

## Materials and methods

### Aim, design, and setting

This study aimed to investigate the effect of prolonged time before analysis and mechanical manipulation on pre-analytical stability and the validity of blood gas analysis results. The Ethics Committee of the Friedrich-Alexander University Erlangen-Nürnberg, Germany reviewed and approved the study (vote 22-124-B). Prospective participants or their legal representatives provided verbal and written informed consent in advance, and all patients participated voluntarily. The study population comprised patients from all surgical specialties who were treated in our interdisciplinary tertiary intensive care unit. Samples were obtained between May 18, 2022 and March 13, 2023. Pregnant women and minors were excluded.

### Blood samples

The blood sampling procedure was standardized, following the syringe manufacturer’s product instructions and in-house standard operating procedures for hygiene and safety. Blood was drawn from indwelling arterial catheters using SafePICO syringes (Radiometer Medical ApS, Brønshøj, Denmark). Immediately after collection, an initial blood gas analysis was performed on an ABL800 FLEX blood gas analyzer (Radiometer Medical) (see Table 1) located in the ICU [5]. The remaining blood in the syringe was not discarded but retained for the study. Each sample syringe was purged of any air bubbles, sealed, and stored on a tray at room temperature next to the analyzer during the 60-minute waiting period. Samples were not shielded from ambient light. Before the second analysis, each syringe was subjected to a standardized mechanical stress protocol: it was dropped ten times from a height of 85 cm (table height) and shaken vigorously ten times. The same sample was then analyzed a second time, 60 minutes after the first analysis. The results of the second analysis were used only for this study and were not reported for clinical decision-making.

**Table 1:** Biomarkers and analyzers, analytical methods, and quality criteria.

Biomarkers	Analyzer model	Manufacturer	Test method	Frequency of quality controls (three shifts per day)	Acceptable root mean standard deviation as defined by Rili-BAEK	Range of interest as defined by Rili- BAEK	Unit
Ca^2+^	ABL800 Flex	Radiometer Medical ApS, Brønshøj, Denmark	Potentiometric method	Once per 8-h shift	14% 7.5%	0.2 to ≤ 1 >1–2.5	mmol/L
Cl^-^	ABL800 Flex	Radiometer Medical ApS, Brønshøj, Denmark	Potentiometric method	Once per 8-h shift	4.5%	70–150	mmol/L
Creatinine	ABL800 Flex	Radiometer Medical ApS, Brønshøj, Denmark	Potentiometric method	Once per 8-h shift	11.5	0.5–10	mg/L
Glucose	ABL800 Flex	Radiometer Medical ApS, Brønshøj, Denmark	Amperometric method	Once per 8-h shift	11%	2.2–22	mmol/L
Hemoglobin	ABL800 Flex	Radiometer Medical ApS, Brønshøj, Denmark	Potentiometric method	Once per 8-h shift	4.0%	2–20	g/dL
K^+^	ABL800 Flex	Radiometer Medical ApS, Brønshøj, Denmark	Potentiometric method	Once per 8-h shift	4.5%	2–8	mmol/L
Lactate	ABL800 Flex	Radiometer Medical ApS, Brønshøj, Denmark	Amperometric method	Once per 8-h shift	11.0%	1–10	mmol/L
Na^+^	ABL800 Flex	Radiometer Medical ApS, Brønshøj, Denmark	Potentiometric method	Once per 8-h shift	3.0%	110–180	mmol/L
pCO_2_	ABL800 Flex	Radiometer Medical ApS, Brønshøj, Denmark	Potentiometric method	Once per 8-h shift	7.5% 6.5%	≤ 35 >35	mmHg
pH	ABL800 Flex	Radiometer Medical ApS, Brønshøj, Denmark	Potentiometric method	Once per 8-h shift	0.4%	6.75–7.80	
pO_2_	ABL800 Flex	Radiometer Medical ApS, Brønshøj, Denmark	Amperometric method	Once per 8-h shift	5.5% 7.0% 11.0%	>125–350 >80 to ≤125 40 to ≤ 80	mmHg
HCO_3_	ABL800 Flex	Radiometer Medical ApS, Brønshøj, Denmark	calculated				
SO_2_	ABL800 Flex	Radiometer Medical ApS, Brønshøj, Denmark	Spectrophotometric method				

For further detailed information on laboratory medical examination methods, we recommend consulting the manufacturer’s user manual and the quality criteria prescribed by the German Medical Association available on the Rili-BAEK homepage (https://www.bundesaerztekammer.de/themen/aerzte/qualitaetssicherung/richtlinien-leitlinien-empfehlungen-stellungnahmen).

### Data analysis and statistics

Bland–Altman plots were used to assess the combined effects of mechanical stress and time delay on the arterial blood gas measurements [6]. Values from the immediate analysis were compared with those from the 60-min delayed, mechanically stressed analysis. Every Bland–Altman plot contains several lines. The central solid line dashed line (shown in red) in the graph represents the mean difference between the two measurements. The two dashed lines above and below the solid line represent the upper and lower limits of agreement (LOA), respectively, corresponding to ±1.96 SD from the mean difference. Typically, 95% of the measured values fell within these limits. As an external criterion for negligible differences, we used the specified allowable error ranges for each analyte according to the German Medical Association’s Rili-BAEK guidelines for quality assurance of medical laboratory examinations [7]. Rili-BAEK defines basic requirements for quality management and acceptable analytical performance in Germany. We assumed that results could be regarded as equivalent (differences negligible) if they lay within the Rili-BAEK error margins [7]. These validity ranges are indicated in the Bland–Altman plots by green lines. The analysis was performed using SPSS (v28.0.1.1; IBM, Armonk, NY) and Microsoft Excel (Microsoft, Redmond, WA). This software was used for statistical computations and plotting, consistent with its use in prior analytical assay studies.

## Results

**Fig 1:**
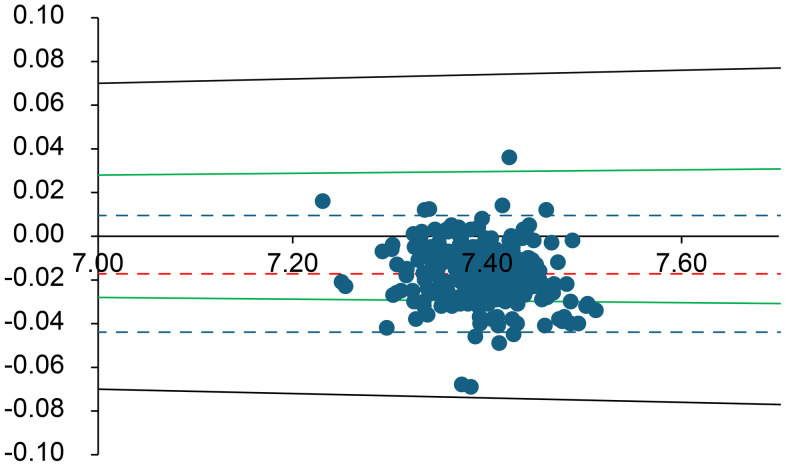
Bland–Altman plot for pH. The difference between the initial and delayed measurements is plotted against their average. The solid red line represents the mean difference (bias), and red dashed lines show the 95% limits of agreement. Green lines denote the Rili-BAEK allowable error range for pH, and black dotted lines mark the ± 10% difference threshold. For pH, the Rili-BAEK quality criterion is only partially met, but all differences remain within a ± 10% margin of the initial value.

**Fig 2:**
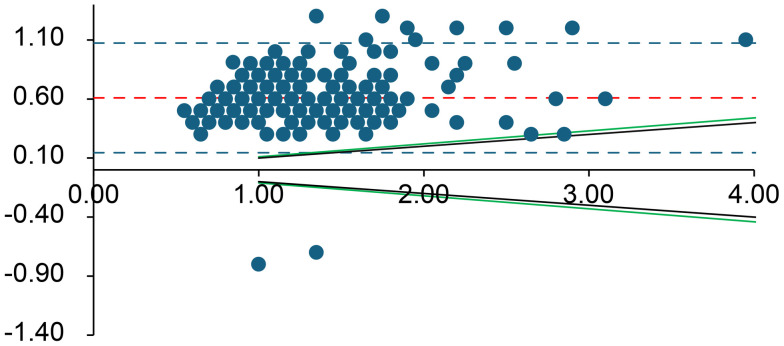
Bland–Altman plot for lactate (mmol/L). The difference between measurements is plotted against their average. Red lines denote the bias (solid) and limits of agreement (dashed); green lines indicate the Rili-BAEK acceptable range; black dotted lines indicate the ± 10% difference threshold. For lactate, the Rili-BAEK allowable error range is not met (delayed values deviate beyond the acceptable limits), and virtually all differences exceed ±10%.

**Fig 3:**
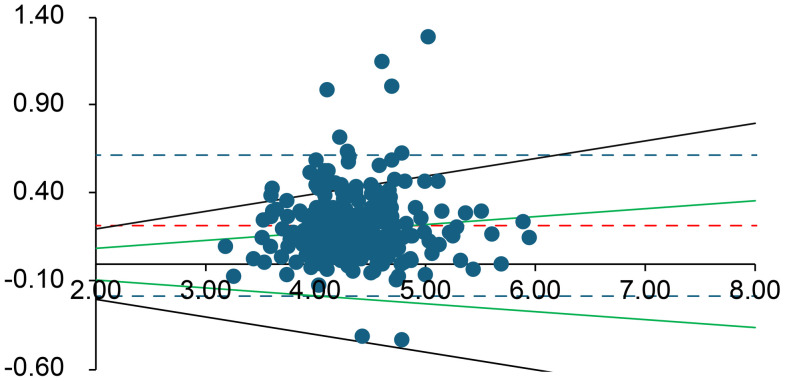
Bland–Altman plot for K⁺ (mmol/L). The difference between measurements is plotted against their average. Red solid and dashed lines show the bias and limits of agreement; green lines show the Rili-BAEK allowable range; black dotted lines show the ± 10% difference threshold. For K ⁺ , the Rili-BAEK quality range is only partially met, but the value deviations remain mostly within a ± 10% margin.

**Fig 4:**
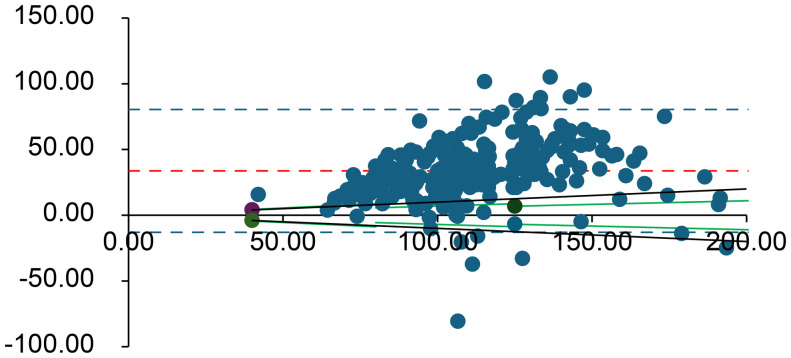
Bland–Altman plot for pO₂ (mmHg). The difference between measurements is plotted against their average. Red lines represent the bias and limits of agreement; green lines represent the Rili-BAEK acceptable range; black dotted lines represent ±10% deviation. For pO_2_, the Rili-BAEK quality criterion cannot be achieved after delay; the observed differences are also usually outside a ± 10% margin of error.

**Fig 5:**
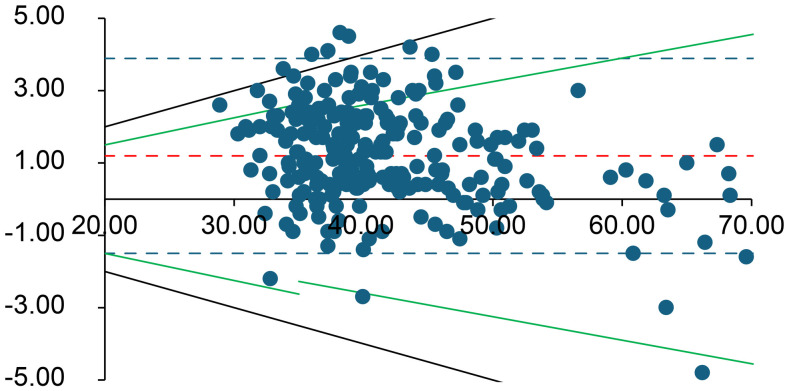
Bland–Altman plot for pCO₂ (mmHg). The difference between measurements is plotted against their average. Red lines indicate the bias and limits of agreement; green lines indicate the Rili-BAEK allowable range; black dotted lines indicate ±10% deviation. For pCO_2_, the Rili-BAEK quality requirement is not met in some cases; however, most differences remain within a ± 10% margin.

**Fig 6:**
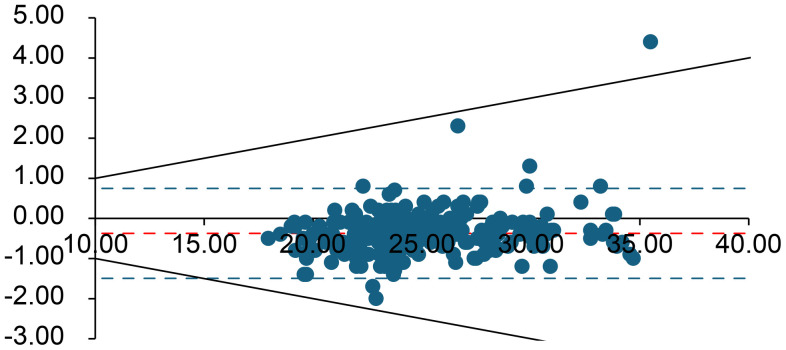
Bland–Altman plot for HCO₃⁻ (mmol/L). The difference between measurements is plotted against their average. Red lines show the bias and limits of agreement; green lines show the Rili-BAEK allowable range; black dotted lines show ±10% difference bounds. For HCO₃ ⁻ , all differences remain within the Rili-BAEK acceptable range. Except for one outlier, the deviations are also within a ± 10% margin of the initial value.

**Fig 7:**
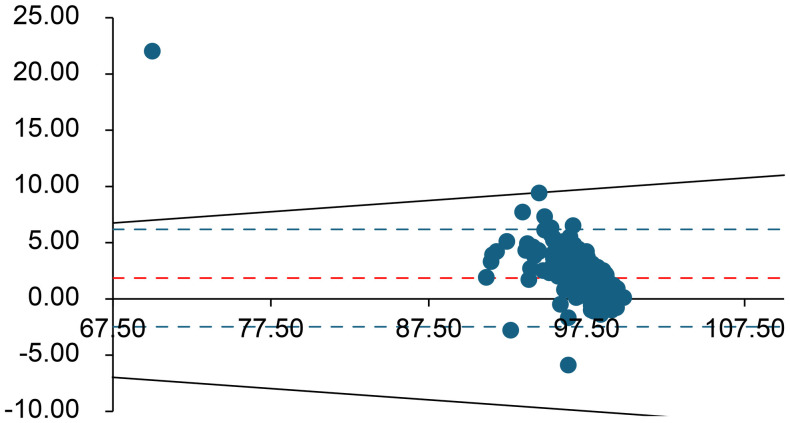
Bland–Altman plot for sO₂ (%). The difference between measurements is plotted against their average. Red lines denote the bias and limits of agreement; green lines (if applicable) would indicate the acceptable range; black dotted lines indicate ±10% difference. For sO_2_, the delayed values remained broadly within acceptable limits, and except for one outlier, the differences stayed within ±10% of the initial measurement.

In total, 240 arterial blood samples were collected. Thirteen analytes were measured, 11 directly measured by the device and 2 derived via calculation. No data were missing. Table 2 summarizes all parameters with their standard deviations and confidence intervals. Each parameter was examined for differences between initial and delayed measurements using Bland–Altman plots. We present here a selection of seven plots in Figs 1–7. Bland–Altman plots for the remaining six parameters are provided as S1–S6 Figs.

**Table 2:** Overview of analyzed biomarkers.

Biomarkers in alphabetical order	Unit	Number of measurements	Mean of differences	CI Mean	Upper limit of agreement	CI Upper limit of agreement	Lower limit of agreement	CI lower limit of agreement
Ca^2+^	mmol/L	240	−0.0073	−0.0074	0.035	−0.0045	−0.049	−0.010
Cl^-^	mmol/L	240	0.104	0.1019	2.836	0.281	−2.628	−0.073
Creatinine	mg/L	239	−0.002	−0.0054	0.162	0.009	−0.166	−0.013
Glucose	mg/L	240	−4.675	−4.8704	4.401	−4.086	−13.751	−5.263
Hemoglobin	g/dL	240	0.098	0.0806	0.518	0.1260	−0.321	0.072
K^+^	mmol/L	240	0.2189	0.2067	0.619	0.245	−0.181	0.193
Lactate	mmol/L	240	0.609	0.6047	1.074	0.639	0.145	0.579
Na^+^	mmol/L	240	−0.077	−0.963	2.465	0.088	−2.619	−0.242
pCO_2_	mmHg	240	1.196	1.2208	1.499	1.372	−3.893	1.022
pH		240	−0.017	−0.0171	0.044	−0.015	−0.009	−0.019
pO_2_	mmol/L	240	33.740	33.7935	80.456	36.771	−12.976	30.709
HCO_3_	mmol/L	240	−0.373	−0.4005	0.748	−0.300	−1.494	−0.4461
SO_2_	%	240	1.855	1.7199	6.184	2.136	−2.475	1.577

Overall, for all analytes *except* K ⁺ , lactate, pCO₂, and pO₂, the differences between the initial and delayed results remained within the Rili-BAEK acceptable error ranges. However, for K ⁺ , lactate, pCO₂, and pO₂, the observed differences often exceeded the Rili-BAEK allowable error. Even a ± 10% deviation threshold (considered as a possible clinical tolerance) was frequently exceeded for these four parameters.

For **potassium (K⁺)** and **pCO**_**2**_, the majority of delayed results remained within 10% of the initial value (88.3% and 97.9% of samples, respectively), although only 52.5% of K⁺ values and 80.4% of pCO₂ values met the strict Rili-BAEK limits. K⁺ increased over time in 93.8% of samples, and pCO₂ increased in 85.4% of samples after the delay.

**Lactate** and **pO**_**2**_ showed the largest deviations after delay. Only 0.4% of delayed lactate measurements remained within the Rili-BAEK acceptable range (likewise, just 0.4% were within ±10% of the initial value), and 99.6% of samples showed higher lactate levels after the 60-minute delay. Similarly, only 5.4% of delayed pO₂ values stayed within Rili-BAEK limits (7.9% were within ±10% of baseline); pO₂ increased in 95% of samples compared to the initial measurement.

Arterial **pH** was relatively stable over 60 minutes. In 84.5% of samples, the delayed pH remained within the Rili-BAEK acceptable range, and *all* samples (100%) stayed within ±10% of the initial pH. pH decreased in 81.6% of samples during the delay (reflecting a slight increase in [H⁺] concentration over time). **Glucose** was also very stable: 98.8% of delayed glucose values remained within the Rili-BAEK error margin, with 89.2% of samples showing a slight decrease in glucose after the delay.

## Discussion

Careful handling and immediate analysis of blood samples are standard practice. However, in a busy and stressful ICU environment, blood samples might unintentionally experience protocol deviations such as analysis delays or agitation during transport. The findings of this study demonstrate that many biomarkers in arterial blood gas samples are relatively robust against a 60-minute analysis delay and substantial mechanical stress. We compared the results of an immediate arterial blood gas analysis to those from the same sample after a defined 60-min delay and simulated mechanical agitation. Notably, we deliberately exaggerated both pre-analytic stressors (delay duration and mechanical force) beyond typical conditions. The rationale was that if certain biomarkers remain stable under this worst-case scenario, they would almost certainly remain stable under more routine pre-analytical conditions.

In our study, the analyzer’s technical accuracy specifications (per Rili-BAEK guidelines) defined what we considered a negligible deviation. We regarded any difference within the known analytical imprecision of the device as clinically equivalent to no change. This objective threshold avoided subjective bias in interpreting changes. We included the most common blood gas analytes in our assessment, so the results can be interpreted on multiple levels.

First, the dissolved gas parameters (pO₂ and pCO₂) underwent significant changes due to diffusion and ongoing biochemical processes during the delay. O₂ diffused into the aterial sample from room air (since the partial pressure of O₂ in ambient air is higher than in blood), causing pO2 in the sample to increase far beyond acceptable limits (on average +38 mmHg, well outside Rili-BAEK ranges). After a 60-min delay, the pO₂ values no longer reflected the patient’s true oxygenation status. Interestingly, we observed a slight net increase in pCO₂ (mean +1.2 mmHg After 60 minutes). At first glance, this seems to contradict the expected net loss of CO₂ from an uncapped syringe. However, the marked rise in oxyhemoglobin in the sample (due to oxygen diffusion) caused bound CO₂ to be released from hemoglobin (the Haldane effect). This CO₂ release apparently outweighed the diffusional losses over the hour, resulting in a net pCO₂ increase in the sample [8–11]. Thus, both gas parameters (especially pO₂) were significantly altered by the delay, rendering them unreliable for clinical interpretation after one hour.

Second, metabolically active constituents such as glucose, lactate, and pH were affected by ongoing cellular metabolism in the stored blood. Red and white blood cells continued to consume glucose and oxygen and produce CO₂ and lactate during the 60-min storage period. Consequently, we saw a systematic decline in glucose concentration (with a corresponding rise in lactate) across nearly all samples (S1 Fig). This negative bias in glucose is well explained by glycolysis; it is well known that blood glucose can decrease over time in vitro as cells metabolize it.Similarly, the accumulation of CO₂ and lactic acid led to a mild decrease in pH on average.. Our findings regarding **lactate** confirm that lactate values cannot be trusted after significant delays: virtually every delayed lactate measurement in our study exceeded even a ± 10% difference, threshold. A lactate measured an hour after sampling is generally not clinically useful, because ongoing anaerobic metabolism in the sample can markedly raise lactate levels [11–13].

With respect to **pH**, we observed only a small average change over 60 minutes (roughly a 0.03–0.07 unit decrease in pH). All delayed pH values corresponded to less than a 10% change in hydrogen ion concentration (i.e., the absolute pH differences were mostly under 0.05 units). However, even such modest pH shifts could be clinically meaningful in critically ill patients. For example, an increase in pH from 7.40 to 7.47 (a 0.07 unit change) represents about a 17% *decrease* in [H⁺]. Thus, while the pH changes in our study fell within our 10% relative threshold (and met analytical performance standards), clinicians should remain cautious—a ~ 10% change in [H⁺] can be significant for patient care. In practice, pH is a crucial parameter that is best measured on a fresh sample; any delayed pH value must be interpreted with appropriate context and caution. Third, the other measured analytes (electrolytes, hemoglobin, creatinine) showed high stability despite the intentional pre-analytical stress. Electrolytes such as Na ⁺ , K ⁺ , Cl ⁻ , Ca² ⁺ , as well as hemoglobin and creatinine, largely remained within both regulatory accuracy limits and exhibited very small mean differences after 60 minutes. These results align with manufacturer data and general laboratory experience, which indicate that many electrolytes and small molecules are relatively stable for about an hour at room temperature. For instance, in our study ~89% of delayed K⁺ results were within 10% of their baseline value. Potassium is generally considered stable for at least 30–60 minutes in heparinized blood at room temperature. Nevertheless, we observed a small subset of cases (about 6% of samples) in which K⁺ increased by more than 10%, with some values exceeding acceptable error margins (Fig 3). This unexpected positive bias in K⁺ likely indicates a pre-analytical artifact. One possibility is mild hemolysis caused by the repeated dropping of the samples, which could release intracellular K from erythrocytes. We did not observe overt hemolysis in the specimens, but micro-hemolysis could have occurred in some cases, leading to elevated K⁺ readings. Another potential factor is prolonged contact of blood with the plastic syringe material; however, known temperature -related effects (e.g., cooling can raise K⁺ by impairingNa ⁺ /K ⁺ -ATPase) do not apply at room temperature. We consider analytical or operator error less likely,though it cannot be entirely excludeed. Overall, given that other studies have also found no significant K⁺ change over 60 minutes at room temperature, we attribute the few outlying K⁺ increases in our data tominor hemolysis or sample handling inconsistencies rather than true instability of potassium [13,14].

Similarly, ionized calcium (Ca²⁺) and the other electrolytes showed minimal average change. A slight decrease in pH could theroretically increase ionized Ca²⁺ (because H⁺ ions displace Ca²⁺ from protein binding sites), but in our results the Ca² ⁺ changes stayed within very tight error bounds, suggesting this effect was negligible. We did observ a small number of samples (see in S2 Fig) with positive biases that exceeded acceptable limits for certain analytes. Such outliers likely stem random analytical variability or minor pre-analytical issues (for example,a sample that was not thoroughly mixed, or a tinyclot affecting one of the measurements). Without additional testing, we cannot definitively explain those few aberrant values. They underscore that even for generally stable analytes, isolated erroneous results can occur, reinforcing the importance of good sample handling and the need to repeat a measurement if a result is clinically implausible.

It is important to emphasize that our findings are not meant to encourage delaying blood gas analyses. Rather, they provide evidence about which parameters are more forgiving of a delay and which are not. In practice, one often does not know how long a sample may have been left at room temperature. Our data suggest that if an arterial sample was inadvertently left for up to an hour, analytes like electrolytes, hemoglobin, creatinine, and glucose would likely still be valid, whereas pO₂ and lactate should be viewed as unreliable under those conditions.

Nonetheless, prompt analysis remains the safest practice, especially for critical blood gas values such as pH, pO₂, and pCO₂. Healthcare staff should be trained to avoid delays in sample analysis whenever possible. If immediate bedside testing cannot be performed (for example, if staff are occupied with other emergencies), one practical approach is to use the blood gas analyzer for the most urgent parameters (the gases and pH) and send the remaining sample to the central laboratory for other, less time-critical tests. This approach would ensure timely results for the crucial measurements while still making use of the sample for additional analyses without requiring an extra blood draw.

## Conclusion

Our study highlights the differential impact of pre-analytical stressors on arterial blood gas analytes. While many measured analytes (e.g., electrolytes, hemoglobin, creatinine) remained stable after a 60-min delay with agitation, pO₂ and lactate values became unreliable. These findings provide evidence-based guidance for clinicians by identifying which delayed blood gas results may still be valid and which cannot be trusted, thereby helping to decide whether a repeat arterial sample is necessary. In practice,selectively avoiding repeat sampling based on specific biomarker stability could reduce unnecessary blood draws, patient discomfort, and healthcare costs without compromising diagnostic accuracy. However, this study was limited to a single-center ICU and an intentionally extreme delay/agitation scenario. The results may differ under shorter delays, different tempearture conditions (e.g., chilled samples on ice), or other clinical settings. Future studies should explore a range of delay times and storage conditions to determine safe windows for sample stability, and should evaluate interventions (such as automated remindersor point- of- care testing strategies) to mitigate pre-analytical errors. In summary, althoug prompt analysis remains the standard to ensure blood gas result accuracy, our findings indicate that certain parameters are relatively robust to a one hour delay, whereas critical indicators of oxygenation and acid-base status require timely measurement. Clinicians can use this information to avoid unnecessary repeat blood draws in some cases, without compromising patient safety.

## Supporting information

S1 Fig Bland–Altman plots for glucose (mmol/L). The difference in measurements is plotted against the average value of both associated measurements. The margins of accuracy, as specified in Rili-BAEK, are drawn as sloping lines. The agreement is high, with random variations, and no systemic bias attributable to treatment was detected. Inaccuracy remains well within the limits outlined in Rili-BAEK and within tolerance of clinical interpretation.
(TIF)

S2 Fig Bland–Altman plots for hemoglobin (g/dL). The difference in measurements is plotted against the average value of both associated measurements. The margins of accuracy, as specified in Rili-BAEK, are drawn as sloping lines. The agreement is high, with random variations, and no systemic bias attributable to treatment was detected. Inaccuracy remains well within the limits specified in Rili-BAEK and the tolerance of clinical interpretation.
(TIF)

S3 Fig Bland–Altman plots for Cl^-^ (mmol/L). The difference in measurements is plotted against the average value of both associated measurements. The margins of accuracy, as specified by Rili-BAEK, are drawn as sloping lines. The agreement is high, with random variations, and no systemic bias attributable to treatment was detected. Inaccuracy remains well within the limits outlined by the Rili-BAEK and tolerance of clinical interpretation.
(TIF)

S4 Fig Bland–Altman plots for creatinine (mg/dL). The difference in measurements is plotted against the average value of both associated measurements. The margins of accuracy, as specified in Rili-BAEK, are drawn as sloping lines. The agreement is high, with random variations, and no systemic bias attributable to treatment was detected. Inaccuracy is well within the limits of the Rili-BAEK and tolerance of clinical interpretation.
(TIF)

S5 Fig Bland–Altman plots for Ca2+ (mmol/L). The difference in measurements is plotted against the average value of both associated measurements. The margins of accuracy, as specified by the Rili-BAEK, are drawn as sloping lines. The agreement is high, with random variations, and no systemic bias attributable to treatment was detected. Inaccuracy is well within the limits of the Rili-BAEK and tolerance of clinical interpretation.
(TIF)

S6 Fig Bland–Altman plots for Na^+^ (mmol/L). The difference in measurements is plotted against the average value of both associated measurements. The margins of accuracy, as specified in Rili-BAEK, are drawn as sloping lines. The agreement is high, with random variations, and no systemic bias attributable to treatment was detected. Inaccuracy is well within the limits of the Rili-BAEK and tolerance of clinical interpretation.
(TIF)
