# Supplementary figures and images for: Stability of arterial blood gas samples after delayed analysis and mechanical stress
Source: PLoS One. 2025 Dec 4;20(12):e0334710. doi: 10.1371/journal.pone.0334710 (PMC12677527; doi:10.1371/journal.pone.0334710)

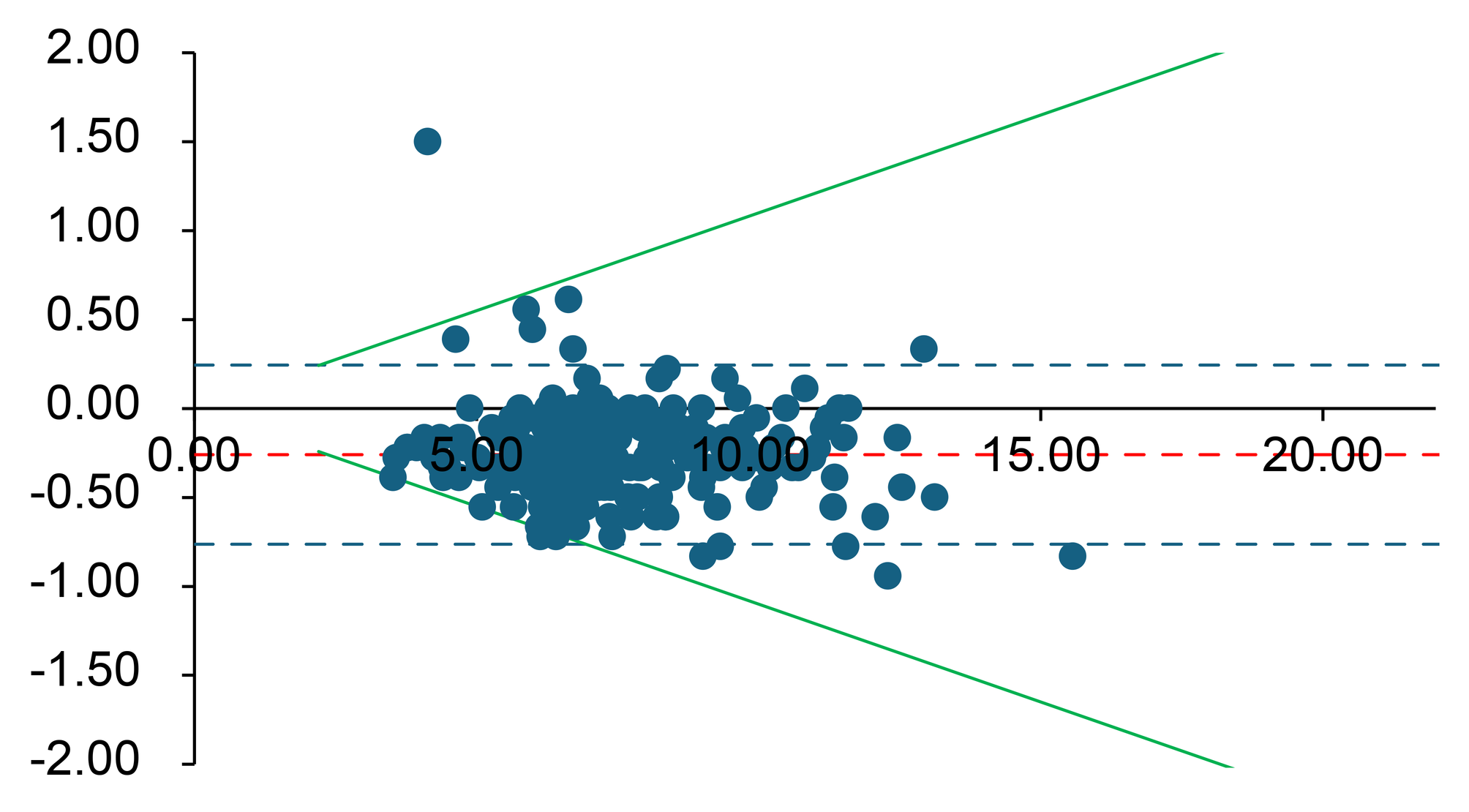

Supplement: S1 Fig — The difference in measurements is plotted against the average value of both associated measurements. The margins of accuracy, as specified in Rili-BAEK, are drawn as sloping lines. The agreement is high, with random variations, and no systemic bias attributable to treatment was detected. Inaccuracy remains well within the limits outlined in Rili-BAEK and within tolerance of clinical interpretation. (TIF) [file pone.0334710.s001.tif]

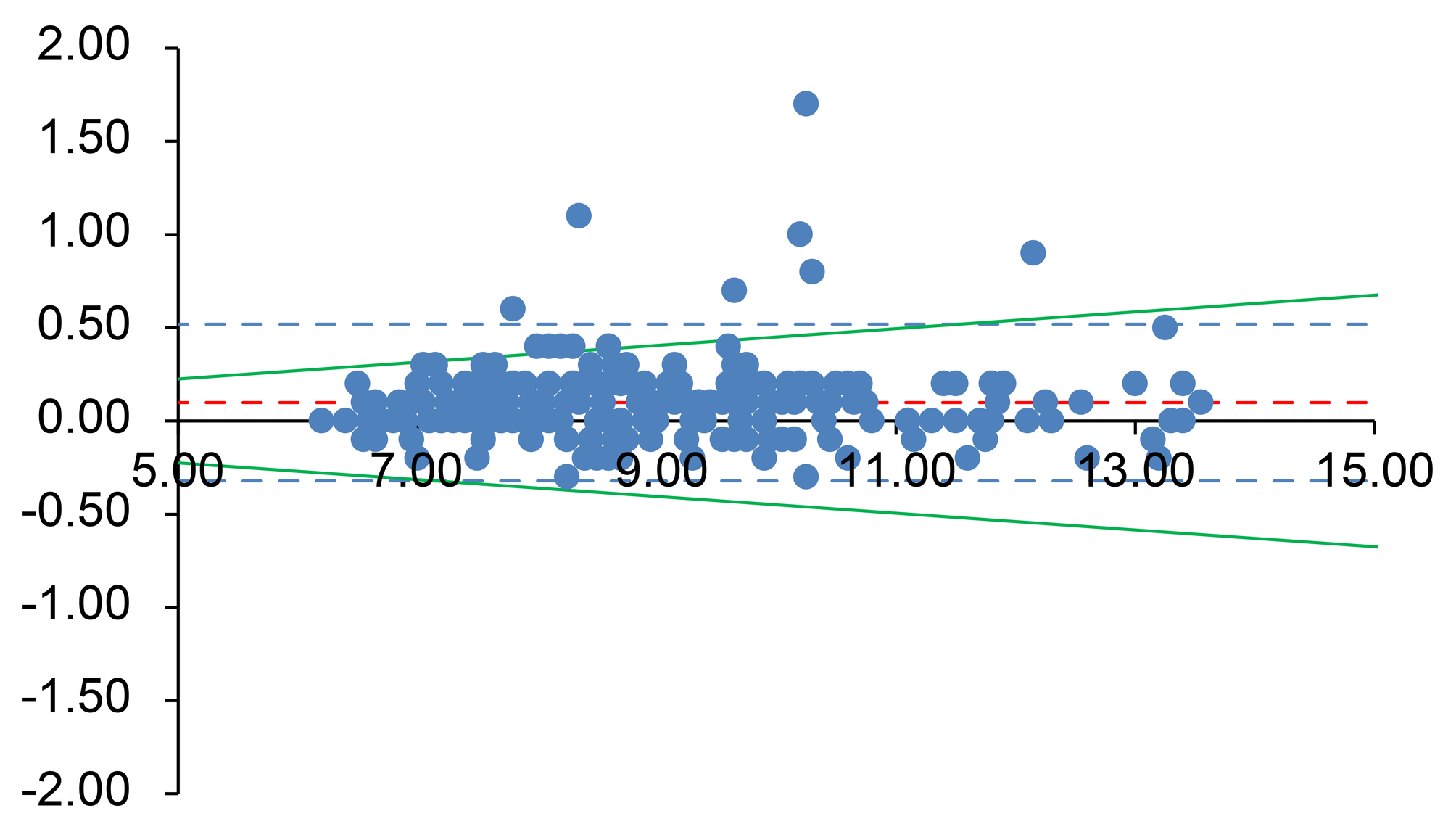

Supplement: S2 Fig — The difference in measurements is plotted against the average value of both associated measurements. The margins of accuracy, as specified in Rili-BAEK, are drawn as sloping lines. The agreement is high, with random variations, and no systemic bias attributable to treatment was detected. Inaccuracy remains well within the limits specified in Rili-BAEK and the tolerance of clinical interpretation. (TIF) [file pone.0334710.s002.tif]

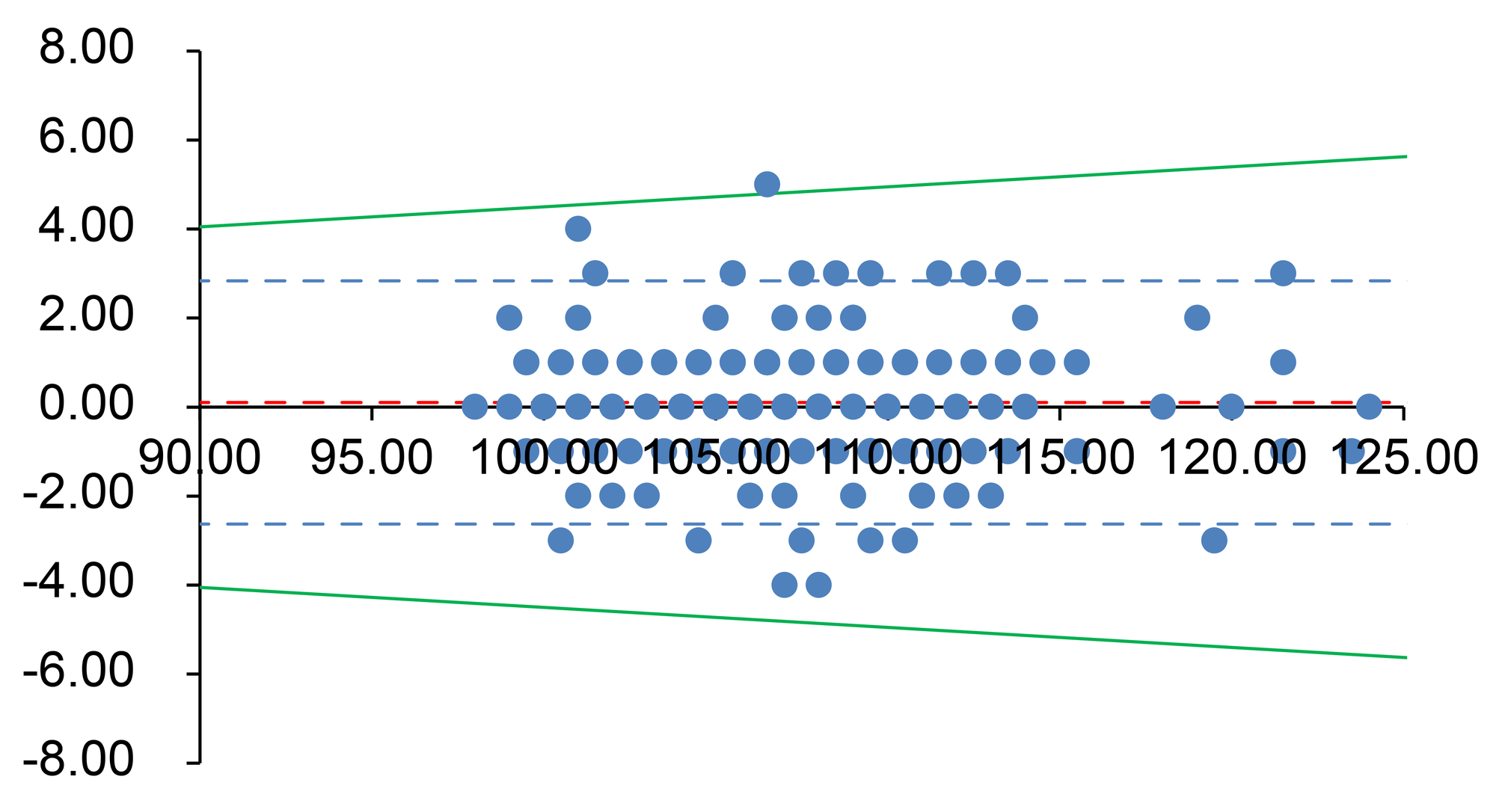

Supplement: S3 Fig — The difference in measurements is plotted against the average value of both associated measurements. The margins of accuracy, as specified by Rili-BAEK, are drawn as sloping lines. The agreement is high, with random variations, and no systemic bias attributable to treatment was detected. Inaccuracy remains well within the limits outlined by the Rili-BAEK and tolerance of clinical interpretation. (TIF) [file pone.0334710.s003.tif]

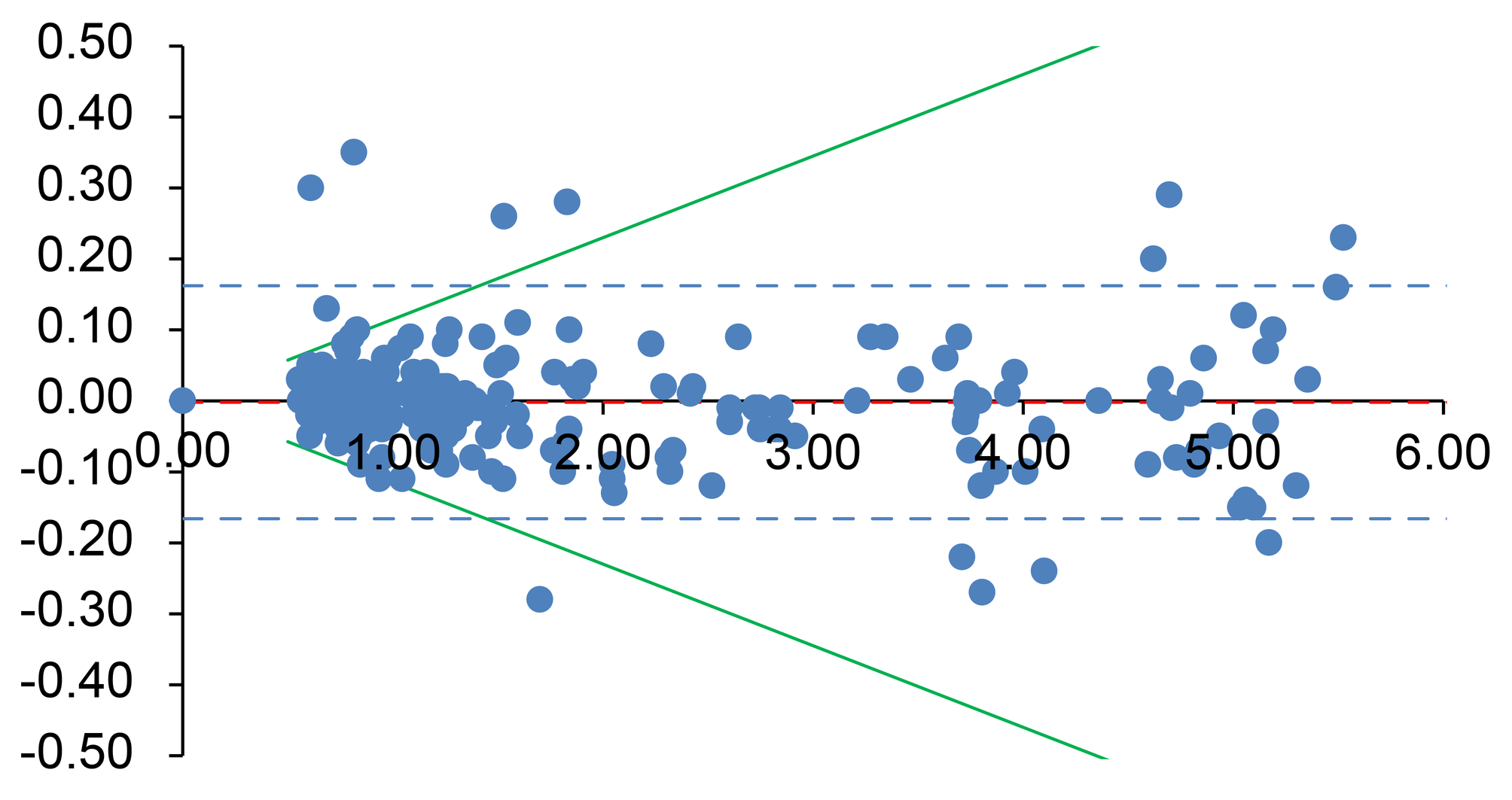

Supplement: S4 Fig — The difference in measurements is plotted against the average value of both associated measurements. The margins of accuracy, as specified in Rili-BAEK, are drawn as sloping lines. The agreement is high, with random variations, and no systemic bias attributable to treatment was detected. Inaccuracy is well within the limits of the Rili-BAEK and tolerance of clinical interpretation. (TIF) [file pone.0334710.s004.tif]

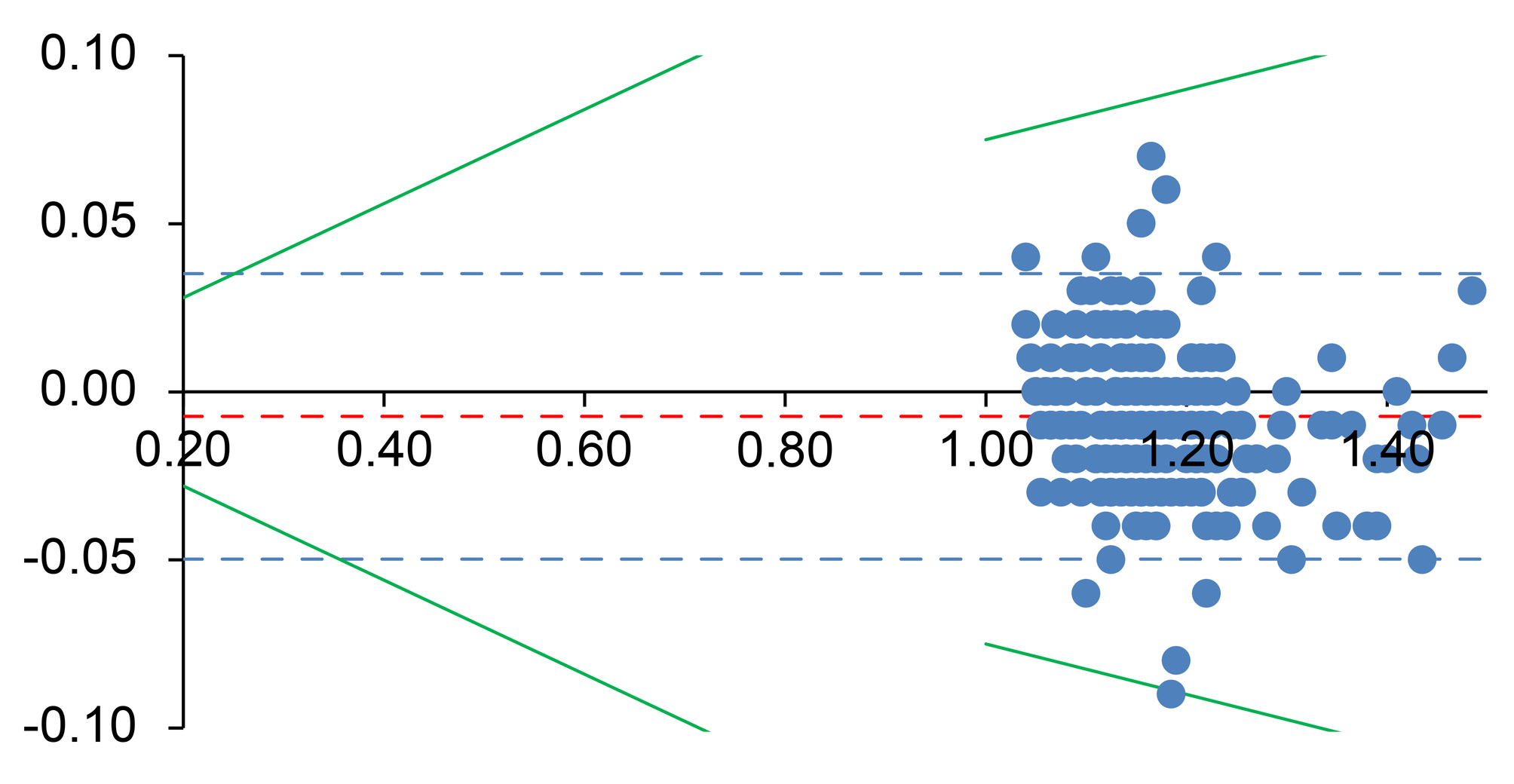

Supplement: S5 Fig — The difference in measurements is plotted against the average value of both associated measurements. The margins of accuracy, as specified by the Rili-BAEK, are drawn as sloping lines. The agreement is high, with random variations, and no systemic bias attributable to treatment was detected. Inaccuracy is well within the limits of the Rili-BAEK and tolerance of clinical interpretation. (TIF) [file pone.0334710.s005.tif]

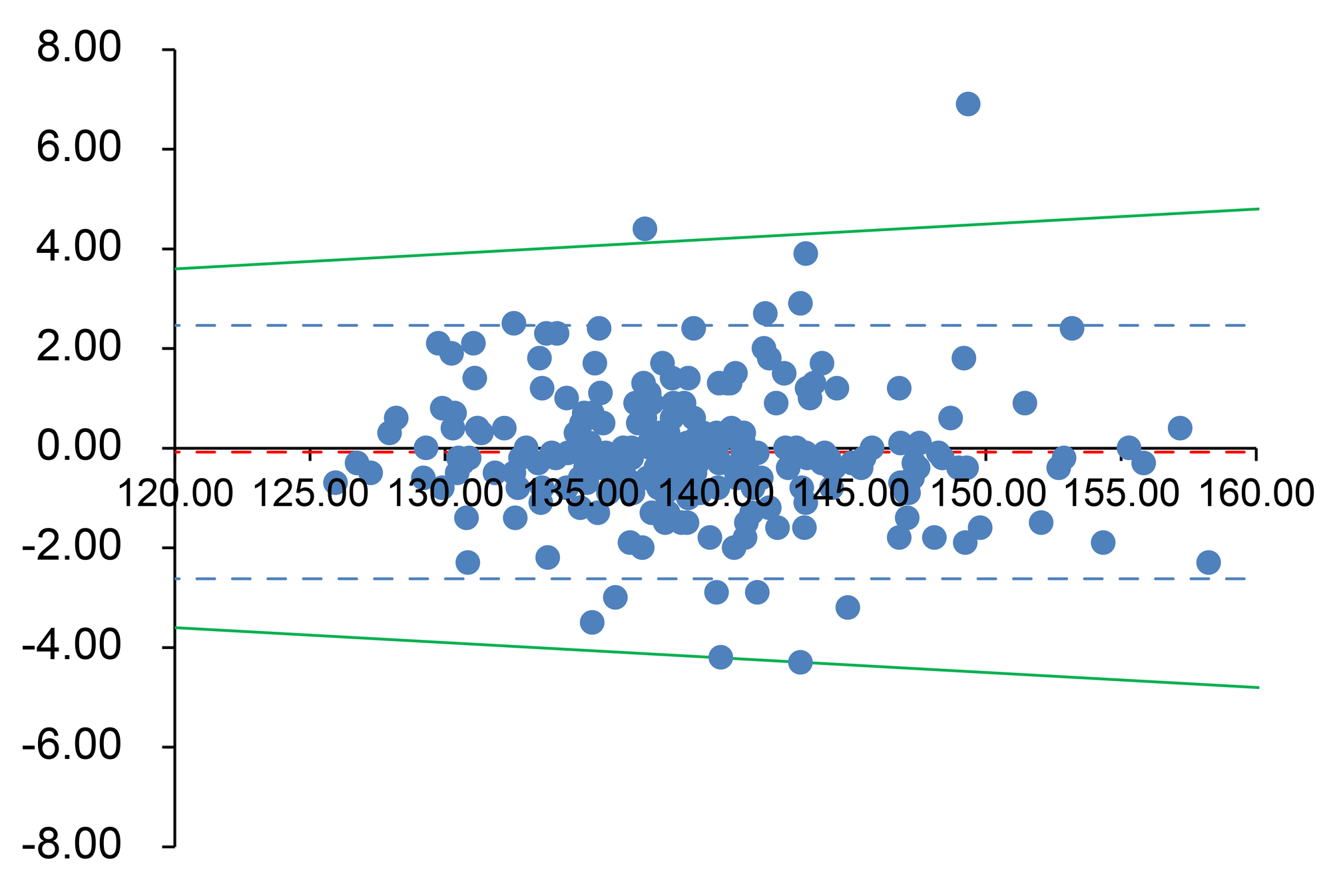

Supplement: S6 Fig — The difference in measurements is plotted against the average value of both associated measurements. The margins of accuracy, as specified in Rili-BAEK, are drawn as sloping lines. The agreement is high, with random variations, and no systemic bias attributable to treatment was detected. Inaccuracy is well within the limits of the Rili-BAEK and tolerance of clinical interpretation. (TIF) [file pone.0334710.s006.tif]
